# The Intricacy of Consuming Fast-Fashion Clothing: The Role of Guilt and Sustainability Values

**DOI:** 10.3390/bs16010138

**Published:** 2026-01-18

**Authors:** Judith Cavazos-Arroyo, Rogelio Puente-Díaz

**Affiliations:** 1 Centro Interdisciplinario de Posgrados, Universidad Popular Autónoma del Estado de Puebla, 21 Sur No. 1103, Col. Santiago, Puebla C.P. 72410, Puebla, Mexico; 2 School of Business and Economics, Universidad Anáhuac México, Av. Universidad Anáhuac 46, Col. Lomas Anáhuac, Huixquilucan C.P. 52786, Estado de Mexico, Mexico

**Keywords:** guilt, fast fashion, values, sustainability

## Abstract

The consumption of clothes creates paradoxes in which values, motives, and emotions interact to generate consumption experiences. To test some of these interactions, we conducted three correlational studies, studies 1, 2, and 3, one experiment, study 4, and one qualitative study, study 5. Study 1 found negative relationships between sustainability values and materialism and positive relationships between sustainable values and the preference for experiential purchases. Study 2 found positive relationships between two components of the slow-fashion movement, equity and exclusiveness, and guilt, and a negative relationship with functionality, another component of slow fashion. Study 3 found an indirect relationship between sustainable values and guilt through their positive and significant relationship with increased awareness of the environmental impact of the fast-fashion industry, supporting a mediation model. Study 4 found that participants were was more likely, regardless of whether the purchase of clothing was labeled as fast fashion or not, to experience pride than guilt when recalling recent past purchases. Last, in study 5, we found that consumers buy clothes to look good and pay attention to quality and value without significant concerns for environmental issues. The implications for consumer behavior were discussed.

## 1. Introduction

Consumption creates a paradox (Olson, 2022). On the one hand, consumption is a ubiquitous activity (Trentmann, 2016) that gives “meaning” to consumers (Miller, 2010). On the other hand, consumers are often reminded of the negative impact of consumption on the environment (E. Ritch, 2020) and asked to reduce their consumption (Olson, 2022; Yan et al., 2015). We posit that this paradox is, in part, created by the multiple values and motives driven consumption (Joy et al., 2012; Mizrachi & Sharon, 2025), which results in a complex set of emotional experiences that sometimes trigger future consumption, while others inhibit it (Kam & Yoo, 2022; Nielsen et al., 2024). We posit that to understand these complex consumer behaviors, we need to establish what the interplay between values, motives, and consumption-related emotions is. To do so, we focus on the consumption of clothes and examine how this paradox unfolds.

Thus, the purpose of the present investigation is to examine the interplay between different sets of values, such as sustainability and orientation toward slow fashion, awareness of the environmental impact of buying clothes from the fast-fashion industry, and the emotional experiences of guilt and pride, in the context of buying clothes from the fashion industry in Mexico. To reach our objective, we rely on affect-as-information and appraisal theoretical approaches and on the importance of values as antecedents of consumers’ actions and conduct five different studies to examine these complex phenomena. We first start with a brief discussion of the context of consumption, followed by discussions of sustainability, affect-as-information, and appraisal theoretical approaches and values, before positing some research questions.

### 1.1. The Context of Consumption

Consumption is a ubiquitous activity across the world (Trentmann, 2016). Historians of consumption suggest that the act of consuming, which entails buying products and experiences, has a rich history dating back hundreds of years (Trentmann, 2012, 2016). Consumption is a dynamic process in which consumers transform material products and experiences in terms of what it means to the self and identity (Miller, 2010). Hence, consumption is construed as more than just purchasing new products or services; it is a reflection of consumers. The act of consumption can be driven by multiple motives, eliciting different meanings, especially in the fashion industry, where clothing is seen as a reflection of consumers’ identity (E. L. Ritch, 2022). Some scholars suggest that the consumption of clothes is mainly driven by appearance motives and that the motive of being sustainable is ambiguous and poorly understood (Joy et al., 2012; E. L. Ritch, 2022). Thus, while sustainability might be a source of concern, appearance, looking good, and enacting different identities through clothing seem to be more powerful motives. Empirical research shows that consumers have a good understanding of sustainability and engage in different sustainable behaviors, such as recycling and consuming less electricity and water, yet they do not see anything wrong with buying clothes from different brands in the fashion industry (Joy et al., 2012; Mizrachi & Sharon, 2025). Similarly, empirical findings showed that brands’ efforts to act sustainably often backfire, leading to more instead of less consumption (Olson, 2022). These findings and the nature of consumption have important implications for understanding the connection between sustainability and the consumption of clothes. In addition, these findings have implications for how consumers appraise (Lazarus, 1991) the act of consuming and buying clothes with different affective consequences.

Specifically, the consumption of clothes is not new. Yet, the concept of the consumption of clothes coming from fast-fashion alternatives is relatively recent and entails a strategy in which brands have short-term cycles of fashion, emphasizing obsolescence (E. L. Ritch, 2022). In addition to the strategy of short-term cycles, brands have limited production of batches of clothes and produce and distribute them efficiently to sell clothes at relatively low prices. What these strategies generate is a focus on novelty, increasing the tendency to see clothes as disposable (Joy et al., 2012). Rapid changes, obsolescence, and a focus on novelty energize a constant search for a new identity. These strategies often lead to overproduction and consumption of clothes (Joy et al., 2012). Overproduction and overconsumption are opposite to the goal of sustainable behavior (Maldini & Klepp, 2025). This is why recent studies have focused on assessing the awareness of the detrimental effect of fast fashion on the environment (Acquaye et al., 2023; Papasolomou et al., 2023; Teerakapibal & Schlegelmilch, 2025). Yet, if consumers do not internalize this problem or are unaware of this connection, they are not likely to experience negative emotions such as guilt. That is, they are not likely to appraise the event of buying clothes from the fashion industry as something negative or detrimental to the environment. We discuss the broad concept of sustainability next, with a special focus on consumption.

### 1.2. Sustainability

Most scholars suggest that we are currently facing the most difficult sustainability challenges of all time (Brem & Puente-Diaz, 2020). Yet, sustainability is a broad, multifaceted concept with many definitions and with different stakeholders. One of the accepted definitions suggests that sustainability involves the current satisfaction of needs by producing and consuming goods that do not jeopardize the satisfaction of the same needs in future generations (E. Ritch, 2020). Experts express alarming warnings about environmental problems and challenges (E. Ritch, 2020). Yet, sustainability is a concept with high causal density, which means that it is affected by multiple variables and actors. In addition, it is a concept used in different contexts and with some degree of ambiguity. The production and consumption of material goods in the form of clothing is one aspect of sustainability. Indeed, the fashion industry, according to a report generated by the United Nations, is the second most polluting industry (United Nations, 2019). Yet, it is not entirely clear whether consumers perceive such a connection. For example, qualitative findings from one study showed that when speaking of sustainability, consumers focused primarily on workers’ rights (Joy et al., 2012).

In addition, sustainability in the production and consumption of clothes involves multiple actors, including government officials, brands, and consumers (Faludi, 2025). Thus, it is a problem with shared responsibility, yet the amount of responsibility that each actor has is not easily determined. We claim that our behavior in consumption contexts and our decisions on sustainability issues are partially informed by emotions. Yet, given the ambiguity of the concept of sustainability (Adolph & Beckmann, 2024), the lack of a clear connection between sustainability and the production and consumption of clothes, at least in the mind of consumers, and the lack of precise assignment of responsibility to each of the actors make it more likely that consumers will experience a wide variety of contradicting emotions that provide different types of information that motivate and inhibit the acquisition of clothes (Kuupole et al., 2025). We now turn our attention to examining emotions as providers of information.

### 1.3. Feeling-as-Information and Appraisal Approaches

Emotions color our lives. Consumption is colored by emotions. We posit that emotions provide information to consumers when engaging in consumption (Clore & Schiller, 2016). Previous work on consumption has focused primarily on positive emotions. Yet, in the last decade, we have seen an increase in the examination of negative emotions in consumption, including the role of guilt in consumer behavior (Antonetti & Maklan, 2014; Cairns et al., 2021). While guilt could come from different reasons, we focus here on the potential guilt experienced from sustainability concerns when buying clothes. Hence, we combine feeling-as-information with appraisal theory and posit that consumers experience conflicting emotions when buying clothes, providing mixed information about the self in the context of consumption.

Feeling-as-information theory poses that subjective experiences, including feelings, emotions, and metacognitive experiences, provide information (Schwarz, 2012). The type of information could be about the situation at hand or the context, and about the self (Clore & Schiller, 2016), a form of self-reflection. We posit that the acts of searching, buying, and wearing clothes elicit different emotions (Andrade, 2015), from satisfaction and pride to guilt. We suggest that these emotions provide different information about the self in the context of consumption with important implications for consumers, brands, and government officials. Yet consumers do not experience the same emotions or with the same intensity. Appraisal theory posits that how individuals appraise a given situation influences the types and intensity of emotions experienced (Lazarus, 1991). Hence, how consumers appraise the act of buying clothes, the connection between buying and producing clothes, and sustainability will, in part, determine the type and intensity of emotions experienced in the context of consumption. One potential factor influencing appraisals is whether the focus of consumption is on replacing clothes or what consumption says about the self (Valor et al., 2022).

Hence, if consumers appraise the act of buying clothes as positive, relevant, and desirable, consumers might experience primarily pride and satisfaction from buying clothes, and the information provided could be construed as a positive indicator of the self (Kam & Yoo, 2022). This positive information is likely to encourage consumers to continue buying clothes frequently. If, on the other hand, consumers appraise the act of buying clothes as negative or damaging, consumers might experience primarily guilt or shame from buying clothes and interpret this information as something negative about the self (Nielsen et al., 2024). This type of negative information could lead to either a reduction in the consumption of clothes or to a shift in focus and preference to brands that help to alleviate this guilt by offering sustainable clothing (Haws et al., 2014). In addition, consumers could resolve their guilt with additional cognitive or behavioral strategies, such as donating used clothes and recycling them (Cairns et al., 2021). Consumers can also “obtain a moral license” by consuming clothes from brands that claim to be “sustainable” (Olson, 2022). Now, we turn our attention to the role of values of antecedents of the appraisals of emotion.

### 1.4. The Role of Values

Values drive and energize behavior (Schwartz, 1973). Specifically, Value–Belief–Norm theory suggests that values play an important role in sustainability (Stern et al., 1999). Yet, as suggested by consumer scholars, consumers can hold multiple values that seem to contradict each other (Cairns et al., 2021; Reczek & Irwin, 2015). Being sustainable or ethical might be increasingly important for consumers, but so is looking good and being frugal (Reczek & Irwin, 2015). Hence, consumers face multiple dilemmas and try to solve them with the available, limited resources they have.

The use of values represents a robust approach to examine sustainability (Phipps et al., 2013), coming from consumption in general and more specifically to the consumption of clothes. For example, some studies assessed the extent to which consumers value products and services that are sustainable (Haws et al., 2014). Other studies focused on assessing the extent to which consumers value a slow-fashion orientation (Jung, 2014), which is conceptualized as the antithesis of the fast-fashion approach to clothes. Both approaches are useful to understand the consumption of clothes and are addressed in our empirical studies.

Thus, the purpose of the present investigation is to examine the interplay between sustainable values, awareness of the environmental impact of buying clothes, and the emotional experiences of guilt and pride in the context of fast-fashion clothing in Mexico. To achieve our research objectives, we use a multi-study strategy, combining correlational and experimental quantitative studies with qualitative in-depth interviews. Our efforts are driven by the following research questions addressed in different studies:

R1: Are sustainability values related to consumption?

R2: Are slow-fashion values, the opposite of fast fashion, correlated with the experience of guilt?

R3: Are sustainability values related to guilt by their connection with awareness of the detrimental effects of the fast-fashion industry?

R4: Do consumers, when recalling recent purchases, experience similar or different levels of guilt as compared to other emotions, such as pride?

R5: When describing recent purchases, do consumers spontaneously report their concern for the environment when buying clothes? Do they spontaneously report experiencing guilt?

### 1.5. Overview of Studies

For our quantitative studies, we followed two guidelines to decide on sample size. First, we used G* Power 3.1 to make sure our studies had a power of 0.95 for detecting small to medium effect sizes (Faul et al., 2007). Second, we collected data in specific, predetermined weeks before starting data collection. For our studies, we collected data from the beginning until the end of a regular semester of 16 weeks. For the sake of space, we do not discuss specific results for each study, but following these two guidelines allows us to have statistical analyses with enough power and have transparency in our data collection. In all five studies, we used samples of convenience, which limited our ability to generalize results. All participants were either recruited from college classes or from popular shopping venues. Yet, in all four quantitative studies, participants were consumers who were actively involved in buying clothes, suggesting that it is a relevant sample to examine.

**Figure 1:**
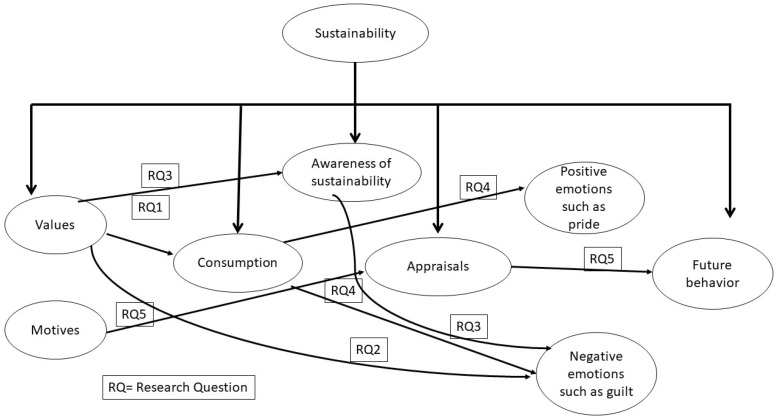
Graphical illustration of five studies and research questions.

Study 1 focused on establishing the relevance of sustainability values in the context of consumption among Mexican consumers. This study was designed to address research question 1. In study 2, we conducted an observational study with a heterogeneous sample of Mexican consumers to test the connection between slow-fashion values and the experience of guilt. This was designed to address research question 2. We then conducted an observational study, study 3, testing a mediation model in which sustainability values are related to guilt through their influence on awareness of how fast-fashion practices are detrimental to the environment. This study addressed research question 3. Fourth, we conducted an experimental study to directly compare the affective experience of guilt coming from buying fast-fashion clothing to address research question 4. Last, we conducted semi-structured qualitative interviews with consumers of clothes to examine questions raised in our previous studies and had a deeper understanding of the connection between different motives for buying clothes and affective experiences. This study was designed to complement our findings and addressed research question 5 (see Figure 1 for a graphic illustration connecting the main construct under investigation with the research questions). All procedures were in accordance with the ethical standards of the human subjects committee of our universities and with the 1964 Helsinki Declaration and its later amendments or comparable ethical standards.

## 2. Study 1

### 2.1. Method of Study 1

#### 2.1.1. Participants

Participants were 298 (194 women, ages 18 to 36 years, M = 22.53 years and SD = 3.17) college students from two universities in Mexico who agreed to participate voluntarily. Participants were frequent consumers of clothes.

#### 2.1.2. Procedure

Participants completed a battery of questionnaires individually. Participation lasted between 6 and 10 min.

#### 2.1.3. Measures

Material Value Scale (MVS) (Richins, 2004): We used the abbreviated nine-item measure of material values. Each item was scored on a scale ranging from 1 to 5, where 1 represented “strongly disagree” and 5 represented “strongly agree.” The scores from the scale had good psychometric properties, especially when it is used to measure materialism at the general level. The coefficient of internal consistency was acceptable, α = 0.81.

Experiential buying tendency scale (Howell et al., 2012): This questionnaire uses four items to measure the tendency to prefer experiential over material buying with a 7-point scale. Scores have shown adequate properties in previous investigations (Howell et al., 2012) and had acceptable levels of internal consistency (α = 0.61).

Sustainable consumption scale (Haws et al., 2014): This questionnaire uses 6 items to assess sustainable values on a scale from 1 (completely disagree) to 7 (completely agree). Scores showed acceptable levels of internal consistency (α = 0.87).

### 2.2. Results of Study 1

Results showed that sustainability values had a negative relationship with materialism, r = −0.194, *p* < 0.001. Conversely, sustainability values had a positive relationship with a preference for experiences over material things, r = 0.214, *p* < 0.001.

### 2.3. Brief Discussion

Our results showed initial support for the role of sustainability values. Sustainability values correlated with consumption-related variables such as materialism and the preference for experiences over material goods in the expected direction, lending evidence for their relevance in consumption contexts. Yet we did not establish the role of consumption-related values. In study 2, we wanted to specifically examine the role of consumption-related values in the experience of guilt by focusing on values coming from the slow-fashion movement.

## 3. Study 2

### 3.1. Method of Study 2

#### 3.1.1. Participants

A total of 400 consumers participated voluntarily in the study without any financial compensation (279 women, 113 men, and 1 individual who self-identified as belonging to a different gender category; 13.5% in the 23–27 age bracket, 24% in the 28–32 age bracket, 25.5% in the 33–37 age bracket, 38–43 37% in the age bracket).

#### 3.1.2. Procedure

A quantitative, non-experimental, explanatory, cross-sectional study was conducted. Data were collected using an electronic survey, with previously validated scales. Participants completed the instrument in approximately 6 to 10 min. All questions were answered in the context of buying clothes from the fast-fashion industry. Thus, all participants reported having bought something from the fast-fashion industry in the past 6 months. While our sample procedure was not probabilistic, this second study had a more heterogeneous sample, which strengthened about design.

#### 3.1.3. Measures

Post-Purchase Guilt (Huang et al., 2022): This questionnaire uses seven items on a scale from 1 (strongly disagree) to 5 (strongly agree). The items were “ Sometimes I feel that the way I buy clothes is impulsive or irrational”, “I feel uneasy or afraid that others might judge my clothing spending habits”, “I avoid showing certain clothes because I fear others will think my purchases are irrational”, “When I stop buying clothes, I feel more stressed or anxious”, “I feel anxious after buying clothes impulsively or without control”, “I feel guilty or ashamed after buying clothes excessively or without restraint”, and “I sometimes regret my behavior when I buy clothes frequently or compulsively”. Scores had acceptable levels of internal consistency (α = 0.94).

Slow-Fashion Orientation (Jung, 2014): This was evaluated across five dimensions—equity (α = 0.818), authenticity (α = 0.826), functionality (α = 0.847), localism (α = 0.801), and exclusivity (α = 0.791)—with three items each on a scale from 1 (completely disagree) to 5 (completely agree). The items for fairness were “Fair compensation for apparel producers is important to me when I buy clothes”, “I am concerned about fair trade when I buy clothes”, and “I am concerned about the working conditions of producers when I buy clothes”. For authenticity, they were “I value clothes made by traditional techniques”, “Craftsmanship is very important in clothes”, and “Handcrafted clothes are more valuable than mass-produced ones”. For functionality, they were “ I often enjoy wearing the same clothes in multiple ways”, “I tend to keep clothes as long as possible rather than discarding quickly”, and “I prefer simple and classic designs”. For localism, they were “I prefer buying clothes made in Mexico to clothes manufactured overseas”, “I believe clothes made of locally produced materials are more valuable”, and “We need to support Mexican apparel brands”. Lastly, for exclusivity, they were “Limited editions hold special appeal for me”, “I am very attracted to rare apparel items”, and “I enjoy having clothes that others do not”.

### 3.2. Results of Study 2

We regressed guilt on five sets of values related to the slow-fashion movement: equity, authenticity, functionality, localism, and exclusiveness. Results showed a significant model, F (5, 399) = 7.52, R^2^ = 0.09. Individual results showed significant positive relationships between guilt and the values of equity and exclusiveness, b = 0.20, *p* = 0.001; b = 0.21, *p* < 0.001, respectively. In addition, the relationship between guilt and functionality was significant and negative, b = 0.20, *p* = 0.008. The relationships of guilt with authenticity and localism were not significant.

### 3.3. Brief Discussion

Results showed that values associated with the slow-fashion movement did play an important role in the experience of guilt. Specifically, the values of equity and exclusiveness had a positive relationship with guilt. Conversely, the relationship between functionality and guilt was significant and negative, suggesting that values were energized by different motives in which the functional component of clothes was important. Functionality might act as a justified reason to buy clothes. One important shortcoming is that we have not integrated into a single model how sustainable values might help individuals be aware of the connection between the fashion industry and sustainability challenges, and whether this might have an impact on the experience of guilt. Another shortcoming is that our previous assessment of guilt included guilt from buying compulsively. Study 3 was designed to address these limitations and research question 3.

## 4. Study 3

### 4.1. Method of Study 3

#### 4.1.1. Participants

Participants were 416 consumers of clothes and shoes (58% women, 73% in the 18–25 age bracket) from Mexico. Participants voluntarily decided to complete a battery of questionnaires without any financial compensation.

#### 4.1.2. Procedure and Measures

The batteries of questionnaires were programmed in Google Forms and administered online. The battery took between 10 and 15 min to complete.

Guilt: Participants rated their feelings on a scale from 1 (I do not feel like that at all) to 10 (I completely feel like that) for the following emotion adjectives: regretful, remorseful, and guilty due to the environmental damage. From these, we calculated one score for guilt by adding the score of each item (α = 0.65). The adjectives were specifically developed for this study, consulting the literature on guilt (Tangney & Dearing, 2002).

Sustainable consumption scale (Haws et al., 2014): We used the same questionnaire as in study 1. Scores showed acceptable levels of internal consistency (α = 0.79).

Fashion industry environmental awareness (Xu et al., 2022): This questionnaire uses three items to assess environmental awareness on a scale from 1 (Does not describe me at all) to 10 (Describes me completely). The items were “I understand the current level of pollution to the environment by the clothing industry”, “I am very concerned about the impact of the clothing industry on environmental development”, and “I think renting second-hand clothing can reduce the production and consumption of clothing, thereby reducing environmental pollution”. Scores showed acceptable levels of internal consistency (α = 0.61).

Responsibility for sustainability in the fashion industry: We asked participants the degree of responsibility for the three main stakeholders in the fashion industry: government officials, clothing and shoe brands, and consumers. Participants were instructed to express the responsibility of each stakeholder as a percentage, so the total sum was 100%.

### 4.2. Results of Study 3

Mplus 7.11 was used to test the latent variable and structural model, employing robust estimation to handle the missing data. A combination of absolute and incremental fit indexes was reported: χ^2^, Root Mean Square Error of Approximation (RMSEA), Comparative Fit Index (CFI), and Tucker–Lewis Index (TLI). The cutoff scores, as the minimum acceptable levels of model fit, were RMSEA ≤ 0.08 and CFI and TLI > 0.90 (West et al., 2012).

We first tested the measurement model with the following latent variables: sustainable consumption values (six items), fashion industry environmental awareness (three items), and guilt (three items). It was important to treat these three variables as latent given that the coefficients of internal were low for two of the variables. Thus, we modeled this measurement error. Results for the measurement model showed an acceptable model fit, χ^2^ = 137.325, *p* < 0.001 (df = 50), RMSEA = 0.065, CFI = 0.924, and TLI = 0.900. Examination of the standardized factor loadings showed that they were all significant and in the expected direction (ranging from 0.44 to 0.72). The latent correlations were of medium size with acceptable levels of discriminant validity (Brown, 2006), ranging from 0.27 to 0.70. Given that the fit of the measurement model was acceptable, the structural model was tested.

Results for the structural model showed an acceptable model fit, χ^2^ = 137.537, *p* < 0.001 (df = 51), RMSEA = 0.064, CFI = 0.924, and TLI = 0.902. Examination of the individual parameters showed a significant relationship between sustainable consumption and fashion industry environmental awareness, γ = 0.707, *p* < 0.001. Similarly, the relationship between fashion industry environmental awareness and guilt was significant, b = 0.352, *p* < 0.001. The indirect relationship between sustainable consumption and guilt through its relationship with environmental awareness was significant, 0.249, *p* < 0.001. Last, the squared multiple correlations for the endogenous variables were fashion industry environmental awareness, 0.50, and guilt, 0.12.

We conducted a one-way repeated-measures Analysis of Variance (ANOVA) to estimate mean differences in assigned responsibility of the detrimental effects of the fast-fashion industry on the environment to government officials, brands of clothes and shoes, and consumers. Results showed a significant effect F = (1,406) 178.93, *p* < 0.001, showing that consumers assigned significantly more responsibility to the government, Mean = 41.96, CI = 40.28, 43.63, SD = 17.17, followed by brands, Mean = 34.28, CI = 32.93, 35.62, SD = 13.78, and consumers, Mean = 24.13, CI = 22.77, 25.48, SD = 13.93

### 4.3. Brief Discussion

Our results showed support for the idea that sustainable consumption values were related to awareness of the detrimental impact of the fashion industry on the environment. This enhanced awareness was positively related to guilt. One potential obstacle to moving consumer behavior to more sustainable practices was that consumers assigned more responsibility to government officials and brands than to consumers. One open question is how consumers experience guilt and other emotions relevant to consumption, such as pride, when recalling their purchases. In addition, another open question is whether labeling a consumption as “fast fashion” leads consumers to experience greater guilt. This fourth study, an experiment, was designed to test these questions and shed additional light on the connection between consumption of fashion and the experience of guilt and pride.

## 5. Study 4

### 5.1. Method of Study 4

#### 5.1.1. Participants

Participants were 204 consumers of clothes (72% women, 69% in the 18–25 age bracket) from two private business schools in Mexico. Participants consented to participate in this study voluntarily without any financial compensation.

#### 5.1.2. Procedure

Participants were randomly assigned to one of the two following conditions:

Labeled as fast fashion condition: In this condition, participants read the following instructions: “Buying clothes or shoes in fast-fashion stores such as H&M, Zara, Shein, Briska, American Eagle, Old Navy, or Pull and Bear makes me feel:”. Hence, in this condition, participants read the fast-fashion label before reporting how they felt.

Not labeled as fast fashion condition: In this condition, participants read the following instructions: “Buying clothes or shoes in stores such as H&M, Zara, Shein, Briska, American Eagle, Old Navy, or Pull and Bear makes me feel:”. Hence, in this condition, participants did not read the fast-fashion label before reporting how they felt. Hence, the only difference between the two conditions was the presence or absence of the fast-fashion label.

#### 5.1.3. Measures

Guilt and Pride: After reading the instructions above, participants rated their feelings on a scale from 1 (I do not feel like that at all) to 10 (I completely feel like that) for the following emotion adjectives: regretful, proud, glad, remorseful, satisfied, and guilty due to the environmental damage. From these, we calculated two scores, one for guilt and one for pride. The composite for guilt was formed by adding scores from regretful, remorseful, and guilty (α = 0.78), as in study 3. The composite for pride was formed by adding scores from proud, glad, and satisfied (α = 0.79). The adjectives were taken from the literature on guilt and pride (Tangney & Dearing, 2002; Williams & DeSteno, 2014).

### 5.2. Results of Study 4

We conducted a mixed Analysis of Variance (ANOVA) with the experimental condition as a between-subject factor with two levels (label versus no label) and emotion ratings as a within-subject factor with two levels as well (guilt and pride). Results showed that the effect of the experimental condition was not significant, F(1, 202) < 1, *p* = 0.75). Similarly, the interaction between the experimental condition and type of emotion was not significant either, F (1, 202) = 2.50, *p* = 0.12). Conversely, results showed a significant effect for the within-subject factor, F (1, 202) = 162.65, suggesting that participants experienced significantly greater levels of pride, M = 6.43, SD = 2.04, than guilt, M = 3.58, SD = 2.22, regardless of whether the label of fast fashion was or was not used to frame their purchase of clothes or shoes (see Table 1 for descriptive statistics for study 1, 2, 3, and 4).

### 5.3. Brief Discussion

Results showed that labeling the act of buying clothing as fast fashion did not significantly influence the amount of guilt or pride experienced by consumers. Our results also revealed that consumers, on average, experienced greater levels of pride than guilt, suggesting that a limited amount of guilt was experienced from buying clothes. This last finding raises the question of what influences the amount of guilt experienced by consumers. It also raises the question of how consumers construe or frame their past purchases. To complement the results from our four previous studies, we conducted personal interviews with consumers. The goal was to assess the extent to which consumers spontaneously expressed concerns about the negative impact of the fashion industry on the environment and the experience of guilt.

## 6. Study 5

### 6.1. Method of Study 5

#### 6.1.1. Participants

A total of 10 participants were recruited through purposive sampling to ensure diversity in age, gender, and clothing consumption patterns. The sample included six women and four men, aged between 24 and 55 years, all residing in urban areas of Mexico. Participants had direct experience purchasing clothing in both physical stores and online platforms. All were familiar with the concepts of fast fashion and slow fashion.

#### 6.1.2. Procedure

This qualitative study employed in-depth interviews to explore participants’ perceptions, consumption habits, and ethical reflections regarding fast fashion and environmentally responsible clothing. Each interview lasted approximately 45 to 70 min and was audio-recorded with prior informed consent.

A semi-structured interview guide was developed to address key topics, including purchase frequency, selection criteria, and consumption motives, awareness of ecological attributes, experiences with sustainable brands, and feelings of guilt or indifference regarding the environmental impact of clothing consumption. The guide allowed for flexibility to probe emergent themes and support spontaneous narrative expression. All interviews were transcribed and anonymized to protect participant confidentiality. Field notes were taken to capture contextual and nonverbal cues. Data collection continued until thematic saturation was reached, as evidenced by the recurrence of discursive patterns and the absence of new relevant codes in the final interviews.

### 6.2. Results of Study 5

Our strategy was first to conduct a microanalysis to identify major categories. Category identification was based on the available research literature and letting the data “speak” (Strauss & Corbin, 1998), combining bottom-up and top-down approaches. The relevant literature allowed us to make sense of verbal responses while trying not to overfit or force-fit the responses into categories. Three major categories were identified: motives to buy clothes, construal or framing of fast-fashion or sustainability concerns, and strategies to reduce dissonance and attribution of responsibility. These categories were consistent with previous themes addressed in the conceptual and empirical literature on sustainability and consumption (Haws et al., 2014; Joy et al., 2012; Olson, 2022; E. L. Ritch, 2022).

#### 6.2.1. Motives to Buy Clothes

Even though there are potentially several motives to energize and direct the purchase of clothing, style and appearance was by far the most robust sub-category. For example, some respondents stated “I pay attention to style, for example, I went to buy clothes for my daughter, and I think about how she is”. “I buy clothes every four months, even without needing them, because I am concerned with my image for work, I need to convey the image of someone who takes care of his look” (male participant).

The second most mentioned sub-category referred to the quality of clothes. For example, one participant stated “Quality, I pay attention to quality, and I do not pay much attention to price”. Some participants mentioned price or value for money. From all ten interviews, there were a few mentions related to sustainability, ecological clothing, or any motives related to the environment or sustainability concerns. Some consumers were aware of more ecological brands, but they did not mention any of these brands spontaneously. They were only mentioned after the interviewer introduced the concepts of sustainability or ecological concerns to the interview.

#### 6.2.2. Construal or Framing of Fast Fashion or Sustainability Concerns

Given that participants did not spontaneously mention any attributes related to sustainability when making purchase decisions, we decided to explicitly ask them about fast fashion and sustainability concerns. Most individuals reported knowing or hearing about the concept. Yet, their construal of these concepts often did not include the act of buying clothes from these types of brands as something negative, undesirable, or controversial. For example, one respondent stated, “I recently decided to buy from Shein”. The interviewer replied, How was the experience? The respondent stated, “Clothes looked different from the images, but the quality was good”.

Most consumers associated fast fashion with mistreatment of workers, without mentioning any concerns with overproduction or consumption, overuse of natural resources, misuse of chemicals, or any concepts related to the environmental footprint of this industry. Buying clothes from fast-fashion brands often did not cause any guilt or remorse, with the exception of occasional mentions of labor rights. Even when the problem of labor rights was expressed, one consumer stated that “it would be worse for these individuals not to have jobs than having poor quality jobs”. It seems then that the participants interviewed experienced low levels of conflict or that they have already worked out this conflict internally by citing some remedies to the problem of fast-fashion clothing.

#### 6.2.3. Strategies to Deal with Conflict or Dissonance

Even though the amount of conflict or dissonance expressed was low, some participants coped with this conflict by using simple strategies. For example, one respondent stated, “I do not feel guilty for not buying eco-friendly clothing, because if I donate some of my clothes, someone else will be able to use them”. Thus, actions such as donating clothes, not buying too much, and using clothes until they wear out helped consumers cope with conflict or dissonance. Thus, the amount of internal conflict coming from buying from fast-fashion brands was low, and multiple strategies were used to cope with this conflict (see Table 2 for a summary of the main results).

### 6.3. Brief Discussion

Consumers want to look good and extract meaning from their purchase of clothes (Joy et al., 2012; Miller, 2010; E. L. Ritch, 2022). Environmental and sustainability concerns did not play an important role in their decisions. The negative associations with fast fashion revolved around labor issues, with limited concern for environmental challenges. When there was some degree of internal conflict, consumers mentioned strategies such as donating clothes, using clothes until they wear out, or trying not to buy too much. These simple strategies appeared to give consumers a moral license to continue buying clothes without experiencing any significant guilt (Olson, 2022).

## 7. General Discussion

The purpose of the present investigation was to examine the interplay between different sets of values, such as sustainability and orientation toward slow fashion, awareness of the environmental impact of buying clothes, and the emotional experiences of guilt and pride in the context of fast-fashion clothing in Mexico. We conducted five studies, combining quantitative and qualitative approaches and different conceptualizations of relevant constructs such as values and guilt, to shed light on five research questions. Our discussion was organized around these five questions.

### 7.1. R1: Are Sustainability Values Related to Consumption?

Study 1 showed that endorsing sustainable values had a negative relationship with materialism and a positive relationship with a preference for experiences over material objects, providing initial evidence for the connection between sustainability and consumption-related variables. In addition, study 3 showed that sustainable values had a positive relationship with awareness of the detrimental environmental impact of the fast-fashion industry. These results were consistent with conceptual developments and empirical studies indicating that sustainable values play an important role in consumer behavior (Haws et al., 2014). Similarly, our results were consistent with studies examining how values drive consumer behavior among young consumers (Yan et al., 2015).

Even though values play an important role (Stern et al., 1999), they might not be enough to deter consumers from buying clothes from the fast-fashion industry. Thus, our next research question focused on the role of values in eliciting feelings of guilt, under the assumption that emotions provide consumers with important information about the self (Clore & Schiller, 2016). If consumers experience high levels of guilt, they might reflect on this emotion and question their behavior.

### 7.2. R2: Are Slow-Fashion Values Correlated with the Experience of Guilt?

The slow-fashion movement, with its respective values, represents the opposite of the fast-fashion orientation. Hence, we found in study 2 that endorsing the value of equity and exclusivity, two components of the slow-fashion movement, was positively related to the experience of guilt. The value of equity refers primarily to workers’ rights. Conceptualizing fast fashion as having a workers’ rights component was also found in our qualitative study. One potential disadvantage is that associating the fast- and slow-fashion movement with workers’ rights primarily prevents consumers from focusing on the environmental component. Results also showed that the value of functionality was negatively associated with guilt. Similar results were observed in our qualitative study. Even among participants aware of the detrimental environmental impact of the fast-fashion industry, functionality played an important role when deciding to buy clothes. Based on our results, we posit that functionality might act as a protective value, allowing consumers to experience less guilt and acting as a justified reason to buy clothes, helping consumers cope with any potential dissonance (Cairns et al., 2021). These results were consistent with a recent study in which brands’ efforts on sustainability gave consumers moral licensing to engage in consumption patterns that were detrimental to the environment (Olson, 2022).

### 7.3. R3: Are Sustainability Values Related to Guilt by Their Connection with Awareness of the Detrimental Effects of Fast Fashion?

Results from study 3 showed that sustainable values were indirectly and positively correlated with the experience of guilt through their positive direct relationship with awareness of the detrimental effect of the fast-fashion industry on the environment. Our results were consistent with conceptual and empirical studies, showing the importance of sustainable values (Haws et al., 2014; Stern et al., 1999). Yet, our study demonstrated that the experience of guilt depended on consumers’ awareness of the connection between certain practices of the fast-fashion industry and sustainability (Cairns et al., 2021). While these results might be encouraging, empirical findings reminded us of at least two potential problems: (1) Consumers’ construal or framing of fast fashion does not always include environmental practices. Instead, it tends to lean heavily on labor rights, as shown in the results of our qualitative study. (2) The concept of eco-friendly practices in clothing, the counterpart of fast fashion, is still an ambiguous concept, preventing widespread endorsement among consumers (Cairns et al., 2021). In addition, recent research has shown that eco-friendly practices, such as using organic cotton and recycled materials, can give consumers a moral license to consume more, potentially offsetting the benefits of buying eco-friendly clothes (Olson, 2022). We posit that these obstacles and the wide range of motives energizing the consumption of clothes might help explain why consumers experience different emotions in the context of consumption. This was addressed in research question four by conducting an experiment.

### 7.4. R4: Do Consumers, When Recalling Recent Purchases, Experience Similar or Different Levels of Guilt as Compared to Other Emotions, Such as Pride?

Results from study 4 showed that consumers experienced significantly greater levels of pride than guilt when recalling past purchases of clothes, regardless of whether these purchases were labeled as fast fashion or not. Similarly, results from our qualitative study showed that most consumers did not mention the experience of guilt as an affective consequence of buying clothes. Thus, the lack of substantial guilt might be a function of several factors: (1) Consumers buy clothes for different reasons (E. Ritch, 2020). If looking good is a more important motive than sustainability concerns, it should not be surprising to observe higher levels of pride than guilt when recalling past purchases. (2) Eco-friendly practices in the fast-fashion industry give a moral license to consumers to buy more and feel less guilt (Olson, 2022). In addition, framing some practices conducted by the fast-fashion industry as environmentally friendly prevents consumers from appraising the fast-fashion movement as detrimental to the environment, consistent with the idea that appraisals trigger specific emotions (Lazarus, 1991). Another potential factor influencing appraisals is whether the focus of consumption is on replacing clothes or what consumption says about the self (Valor et al., 2022). Focusing on replacing clothes, buying new clothes, can lead to feeling proud, while focusing on what consumption patterns of clothes says about the self can lead to guilt. Given these results and the complexity of the fast-fashion movement and consumers’ affective responses, we conducted an additional qualitative study to shed light on this important phenomenon.

### 7.5. R5: When Describing Recent Purchases, Do Consumers Spontaneously Report Their Concern for the Environment When Buying Clothes? Do They Spontaneously Report Experiencing Guilt?

Our qualitative findings shed light on some of the intricacies of buying clothes. Consumers bought clothes for different reasons, style and quality being two of the main drivers. Consumers did not mention any environmental concerns. This was consistent with previous findings (E. Ritch, 2020). When explicitly asked about the fast-fashion industry, consumers focused primarily on labor issues, consistent with previous studies (Cairns et al., 2021) and with some of the empirical results of Study 2.

When consumers were explicitly asked about the fast-fashion industry, some concerns were raised. Yet, consumers coped with this potential conflict or dissonance by using relatively simple strategies. For example, consumers expressed that they do not buy “too much”, donate used clothes, and use clothes until they wear out to cope with any potential conflict between personal values and some of the practices of the fast-fashion industry. These simple arguments appear to give consumers a moral license to consume (Olson, 2022).

### 7.6. Limitations

These studies had several limitations. First, none of our designs followed consumers over time, limiting our ability to understand, for example, how sustainable values or the experience of guilt unfold over time. Future studies should employ longitudinal designs. Second, our studies used samples of convenience, mainly college students, trying to cover a wide range of ages, limiting our ability to generalize the results to the entire population of Mexico. Future studies should use probabilistic sampling. Third, we used different conceptualizations of values and guilt to conduct our studies. While this could be a strength, it is a limitation as well.

In sum, across five studies, we were able to show that (1) sustainable values were connected with consumption-related variables; (2) some values coming from slow-fashion perspectives increased the probability of experiencing guilt; (3) sustainable values were indirectly related to guilt through their relationship with the awareness of the detrimental effects of the fast-fashion industry on the environment; (4) given the multiple motives to buy clothes, consumers experienced significantly greater pride than guilt when recalling a recent purchase of clothes; and (5) consumers expressed little concern over the environment when buying clothes and experienced low levels of guilt. When consumers experienced some degree of internal conflict, they relied on simple strategies to alleviate this discomfort. As stated at the beginning of our article, consumption creates interesting paradoxes with implications for policymakers and business practitioners. We tried to capture this by conducting five different studies. While the take-home message might not be crystal clear, it sure ignites our desire to continue conducting research. We hope this enthusiasm is shared by our fellow researchers.

## Figures and Tables

**Table 1:** Descriptive statistics for studies 1, 2, 3, and 4.

		Mean	SD	Range
Study 1	Sustainability values	5.35	1.17	6.00
	Material values	2.76	0.67	3.89
	Preference for experiences	4.65	1.14	6.00
Study 2	Guilt	2.24	0.89	4.00
	Equity	3.16	0.92	4.00
	Authenticity	3.61	0.99	4.00
	Functionality	3.78	1.02	4.00
	Localism	3.42	0.91	4.00
	Exclusivity	3.02	1.07	4.00
Study 3	Guilt	4.63	1.91	9.00
	Sustainability values	5.55	1.82	9.00
	Awareness of detrimental effects	5.49	1.89	9.00
	Res. government officials	42.09	17.31	90
	Res. brands	34.28	13.78	90
	Res. consumers	24.13	13.93	90
Study 4	Guilt	3.58	2.22	9.00
	Pride	6.43	2.04	9.00

**Table 2:** Thematic synthesis of qualitative findings.

Category	Key Interpretive Findings	Value–Emotion Interplay
Motives to buy clothes	Style and appearance Quality Price–value relationship Sustainability is overlooked	Aesthetic and functional values Sustainability values remain inactive and do not generate emotional conflict
Construal or framing of fast fashion or sustainability concerns	Concern for labor rights Environmental impact is rarely mentioned Fast fashion hardly ever triggers guilt	Fast fashion is not perceived as environmentally harmful Ethical concerns are minimized Guilt is rare and linked more to social justice (e.g., labor conditions) than ecological impact.
Coping strategies and attribution of responsibility	Donation Prolonged use Purchase moderation	Dissonance resolved through personal ethics (e.g., donation) to continue buying clothes without experiencing any significant guilt

## Data Availability

All data sets are available upon request from the first author.
